# BET bromodomain inhibitor (JQ1) and tumor angiogenesis

**DOI:** 10.18632/oncoscience.326

**Published:** 2016-11-23

**Authors:** Hemant Kumar Bid, Samuel Kerk

**Affiliations:** Life Sciences Institute (LSI), University of Michigan (UM), Ann Arbor, MI, USA

**Keywords:** angiogenesis, JQ1, bromodomain, MYC

Angiogenesis is one of the most critical multistep biological essentials affecting the development and progression of cancer. It has been explored for decades as a potential target for therapy after endless preclinical and clinical studies. Currently, conceptually promising FDA-approved agents, such as bevacizumab (Avastin, Genentech/Roche), sorafenib (Nexavar, Bayer), and sunitinib (Sutent, Pfizer), have twisted only modest effects in the clinic and do not result in lasting responses of cancer treatment [1]. Tumors have proven to be either intrinsic resistant or acquired resistance through evasion via mutation or recruitment of surplus pro-angiogenic factors [1]. Molecular targeted therapies comprising anti-antiangiogenic potential are becoming more widely accepted in drug discovery era as compared to established anticancer treatment approaches and have more promising results in numerous types of cancers.

JQ1, a bromodomain inhibitor produced by James Bradner, (Tensha Therapeutics acquired by Roche) has direct antitumor and antiangiogenic properties. This small molecule inhibitor targets BRD4, a member of the bromodomain and extra-terminal (BET) family of transcription factors. BRD4 binds to acetylated lysine residues within chromatin, and recruits positive transcription elongation factor (P-TEFb) and other super enhancers involved in transcription. JQ-1 prevents the BRD4-acetylated lysine interaction by competitively binding to BRD4 and inhibiting transcription. In multiple myeloma (MM), a disease frequently associated with dysregulated BET activity, a direct interaction between BRD4 and IgH enhancers located within the *MYC* locus was observed. JQ1 prohibited this interaction, suppressed *MYC* transcription, and reduced the levels of downstream effectors. JQ1 treatment induced cell senescence and apoptosis in multiple MM cell lines, and slowed tumor growth and in orthotopic MM mouse models leading to increased survival [2].

The ability of JQ-1 to inhibit *MYC* transcription has important implications in angiogenesis via blocking VEGF, notch pathway, etc (Figure 1). One study observed that c-Myc knockout mice displayed dysfunctional endothelial cell activity and impaired vascular development in embryonic yolk sacs. Furthermore, the loss of c-Myc reduced the tumorogenicity and differentiation ability of embryonic stem (ES) cells. Re-introducing VEGF reversed the effects of c-Myc knockout. C-Myc also increased the expression of other pro-angiogenic factors such as angiopoietin-2 (ANG-2) and down-regulated anti-angiogenic factors like ANG-1 and thrombospondin-1 (TSP-1) [3]. Indeed, in a study with a transgenic mouse model of Myc oncogenesis, overexpressing Myc in pancreatic β cells quickly increased the expression of the inflammatory cytokine IL-1β, activating matrix metalloproteases (MMP) that in turn released VEGF-A sequestered in the extracellular matrix (ECM). VEGF-A localized to its receptor Flk-1 on the surrounding endothelial cells, promoting their proliferation [4]. It can be inferred from these studies that the ability of JQ-1 to inhibit *MYC* transcription is one mechanism by which it can target angiogenesis in developing tumors.

**Figure 1:**
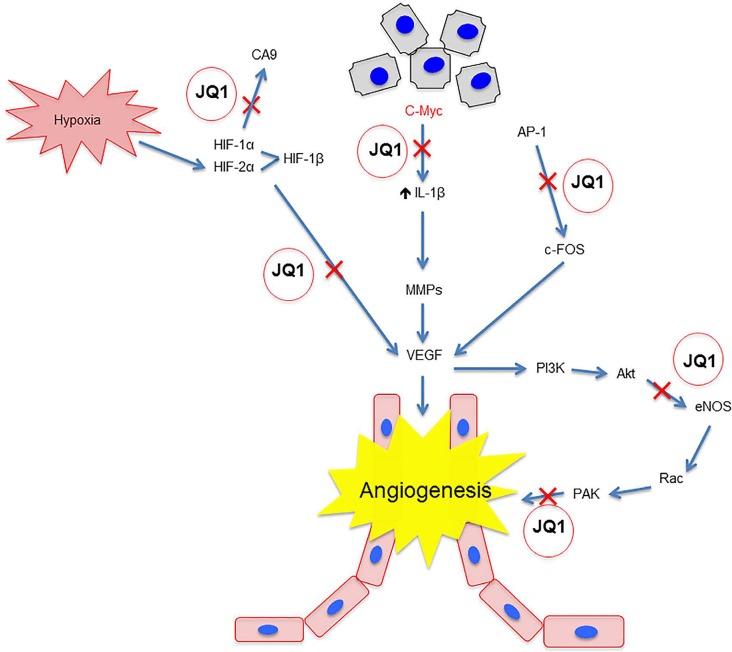
JQ1 blocks angiogenesis via multiple mechanisms.

Other studies have shown anti-angiogenic activity of JQ-1 via c-Myc-independent pathways. The first study to implicate JQ-1 as an agent in angiogenesis conducted by Bid et al 2016 showed that in various models of various childhood sarcomas, JQ-1 inhibited proliferation and angiogenesis irrespective of c-Myc levels both in cell lines and in sarcoma xenografts. Interestingly, while JQ1 had a modest effect on c-Myc in HUVEC cells associated with these sarcoma tumors levels, it significantly suppressed VEGF-induced differentiation, invasion, and viability through a dramatic reduction in AP-1 and FOSL1 levels, which are factors involved in regulating angiogenesis and invasion in endothelial cells [5]. Another study showed that JQ-1 reversed hyper-permeability commonly associated with VEGF activity, and prevented migration and cytoskeletal rearrangements in HUVECS. JQ1 accomplished this through two mechanisms. First, it interrupted the VEGFR2-mediated activation of p21-activated kinase (PAK1), a factor associated with endothelial cell migration, permeability, and angiogenesis. Second, JQ-1 prevented VEGFR2 from activating endothelial nitric oxide synthase (eNOS), which is also important in blood vessel permeability [6]. JQ-1 also showed an ability to prevent cancer cell evasion of angiogenic treatment through the hypoxic response. Under hypoxic conditions, hypoxia inducible factor (HIF) increases the expression of CA9 in order to regulate pH within the tumor, and VEGF to stimulate new blood vessel formation. JQ-1 down-regulated hypoxic response genes and prevented HIF from binding to the CA9 and VEGF promoters in a model of triple negative breast cancer [7].

These studies demonstrated that JQ1 has potential to overcome both the evasive and inherent resistance faced by current therapies that target tumor angiogenesis. It can act on a broad scale across a range of diverse malignancies, whether by inhibiting c-Myc activity, preventing tumor-associated endothelial cell activation, or abrogating the hypoxic response. The multi-faceted inhibitory function of JQ-1 suggests further study regarding its efficacy either alone or in combination with chemotherapy and other therapeutic agents in targeting immune system.

## References

[1] Bergers G (2008). Nat Rev Cancer.

[2] Delmore JE (2011). Cell.

[3] Baudino TA (2002). Genes Dev.

[4] Shchors K (2006). Genes Dev.

[5] Bid HK (2016). Mol Cancer Ther.

[6] da Motta LL (2016). Oncogene.

[7] Huang M (2016). Sci Rep.

